# Transcriptomic and metabolomic changes might predict frailty in SAMP8 mice

**DOI:** 10.1111/acel.14263

**Published:** 2024-07-03

**Authors:** Letizia Dacomo, Pietro La Vitola, Laura Brunelli, Letizia Messa, Edoardo Micotti, Luisa Artioli, Elena Sinopoli, Greta Cecutti, Susanna Leva, Stella Gagliardi, Orietta Pansarasa, Stephana Carelli, Antonio Guaita, Roberta Pastorelli, Gianluigi Forloni, Cristina Cereda, Claudia Balducci

**Affiliations:** ^1^ Department of Neuroscience Istituto di Ricerche Farmacologiche Mario Negri IRCCS Milan Italy; ^2^ Department of Environmental Health Sciences Laboratory of Metabolites and Proteins in Translational Research, Istituto di Ricerche Farmacologiche Mario Negri IRCCS Milan Italy; ^3^ Department of Electronics Information and Bioengineering (DEIB) Politecnico di Milano Milan Italy; ^4^ Department of Pediatrics, Center of Functional Genomics and Rare Diseases Buzzi Children's Hospital Milan Italy; ^5^ Molecular Biology and Transcriptomics IRCCS Mondino Foundation Pavia Italy; ^6^ Cellular Model and Neuroepigenetics IRCCS Mondino Foundation Pavia Italy; ^7^ Department of Biomedical and Clinical Sciences, Pediatric Clinical Research Center “Romeo Ed Enrica Invernizzi” University of Milan Milan Italy; ^8^ Golgi Cenci Foundation Milan Italy

**Keywords:** aging, dementia, frailty, motor impairment, neuronal loss, predictive biomarkers, senescent SAMP8 mice

## Abstract

Frailty is a geriatric, multi‐dimensional syndrome that reflects multisystem physiological change and is a transversal measure of reduced resilience to negative events. It is characterized by weakness, frequent falls, cognitive decline, increased hospitalization and dead and represents a risk factor for the development of Alzheimer's disease (AD). The fact that frailty is recognized as a reversible condition encourages the identification of earlier biomarkers to timely predict and prevent its occurrence. SAMP8 (Senescence‐Accelerated Mouse Prone‐8) mice represent the most appropriate preclinical model to this aim and were used in this study to carry transcriptional and metabolic analyses in the brain and plasma, respectively, upon a characterization at cognitive, motor, structural, and neuropathological level at 2.5, 6, and 9 months of age. At 2.5 months, SAMP8 mice started displaying memory deficits, muscle weakness, and motor impairment. Functional alterations were associated with a neurodevelopmental deficiency associated with reduced neuronal density and glial cell loss. Through transcriptomics, we identified specific genetic signatures well distinguishing SAMP8 mice at 6 months, whereas plasma metabolomics allowed to segregate SAMP8 mice from SAMR1 already at 2.5 months of age by detecting constitutively lower levels of acylcarnitines and lipids in SAMP8 at all ages investigated correlating with functional deficits and neuropathological signs. Our findings suggest that specific genetic alterations at central level, as well as metabolomic changes in plasma, might allow to early assess a frail condition leading to dementia development, which paves the foundation for future investigation in a clinical setting.

## Graphical abstract

SAMP8 frail mice display progressive cognitive and motor dysfunctions from 2.5 to 9 months of age, associated with brain damages. At 2.5 and 6 months, brain transcriptomics identified specific genetic signatures and de‐regulated pathways. Plasma metabolomics detected constitutively lower levels of acylcarnitines and lipids in SAMP8 already at 2.5 months of age, correlating with functional deficits and neuropathological signs at the ages investigated.

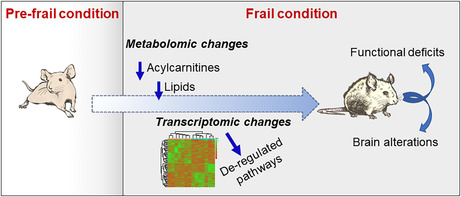

**Abbreviations**
ACs: acylcarnitines
AD: Alzheimer's disease
CNS: central nervous system
DE: differentially expressed
DG: dentate gyrus
DI: discrimination index
FLASH: fast low‐angle shot magnetic resonance imaging
GSEA: gene set enrichment analysis
KEGG: kyoto encyclopedia of genes and genomes
LC: liquid chromatography
lysoPCs: lysophosphatidylcholines
MCR: motoric cognitive risk
MRI: magnetic resonance Imaging
MRM: multiple reaction monitoring
MS/MS: tandem mass spectrometry
N4BiasFieldCorrection: nonuniform bias field correction
NORT: novel object recognition test
PCA: principal component analysis
PCs: phosphatidylcholine
SAMP8: senescence‐accelerated mouse prone‐8

## 1 INTRODUCTION

The world's older population has grown at an unprecedented pace, and according to the World Health Organization, approximately 2 billion people will be older than 65 years in 2050. Along with the worldwide population aging, the number of people living with age‐related diseases has increased dramatically (Li et al., 2021). Among others, Alzheimer's disease (AD)—the most diffused form of dementia—has become a clinical and public health priority, affecting nearly 45 million people worldwide (Mucke, 2009; Prince et al., 2013; Scheltens et al., 2021).

Frailty, a clinic geriatric syndrome characterized by increased vulnerability to stressors due to loss of biological reserves, is becoming the more common challenge as populations age and life expectancy lengthens and represents a possible risk factor for the development of AD in its sporadic form, accounting for approximately 90% of AD cases (Guerreiro & Hardy, 2014), and for other dementia (Wallace et al., 2019; Ward et al., 2022). Current evidence suggests a wide range of frailty prevalence in adults aged ≥65 years living with dementia in both acute care and community settings (Koria et al., 2022). Of note, frailty, which accumulates over time, is nowadays recognized as a reversible condition (Clegg et al., 2013; Rodriguez‐Mañas & Fried, 2015), which implies that recognizing fundamental frailty‐related biological processes and predictive biomarkers will allow to timely predict and prevent its occurrence. To date, very few studies have tried to explain how frailty occurs at the level of molecules, cells, and tissues. The enhanced number of frailty‐related biomarkers and multisystem dysregulation in frailty syndrome (Wang et al., 2019) makes, indeed very difficult to highlight specific determinant factors. Longitudinal research examining multiple biomarkers and frailty‐related outcomes is thus urgently needed.

Based on these considerations, in the present study we attempted to identify frailty‐related biomarkers and genetic signature changes throughout aging in the Senescence‐Accelerated Mouse Prone‐8 (SAMP8) mouse model. This spontaneous model is the most widely used at preclinical level to study frailty associated with the development of an AD‐like dementia (Liu, Liu, & Shi, 2020; Peng et al., 2018; Takeda, 1999). To reach our goals, SAMP8 and their relative control SAMR1 mice (Senescence‐Accelerated Mouse Resistant‐1) underwent a wide behavioral characterization at 2.5, 6, and 9 months of age, at both cognitive level and motor level. At each time point, following behavioral tests, mice were analyzed through structural magnetic resonance imaging (MRI) to assess brain volumetric changes. Subsequently, brain areas such as the cortex and the hippocampus from one hemisphere were collected and analyzed through transcriptomics, while the other hemisphere was analyzed through immunohistochemistry to define the extent of neuropathology in terms of cell density and neuroinflammation. Plasma samples were collected for metabolomics analysis.

We found that, as early as 2.5 months, SAMP8 mice displayed memory deficits and muscle weakness, associated with a neurodevelopment defect in the cortex, hippocampus, striatum, and whole brain, associated with a progressively lower neuronal density in all areas, transient astrogliosis, and progressive loss of microglial cell. Transcriptomic brain analysis found significant differences in gene expression at 2.5 months, which became even greater at 6 months, with pathway analysis highlighting changes attributable to SAMP8 cognitive and motor deficits. Furthermore, plasma metabolomics unveiled at 2.5 months a significant constitutive lower level of acyl‐carnitines and lipids in SAMP8 mice.

## 2 RESULTS

### 2.1 Cognitive dysfunctions in SAMP8 mice

**FIGURE 1:**
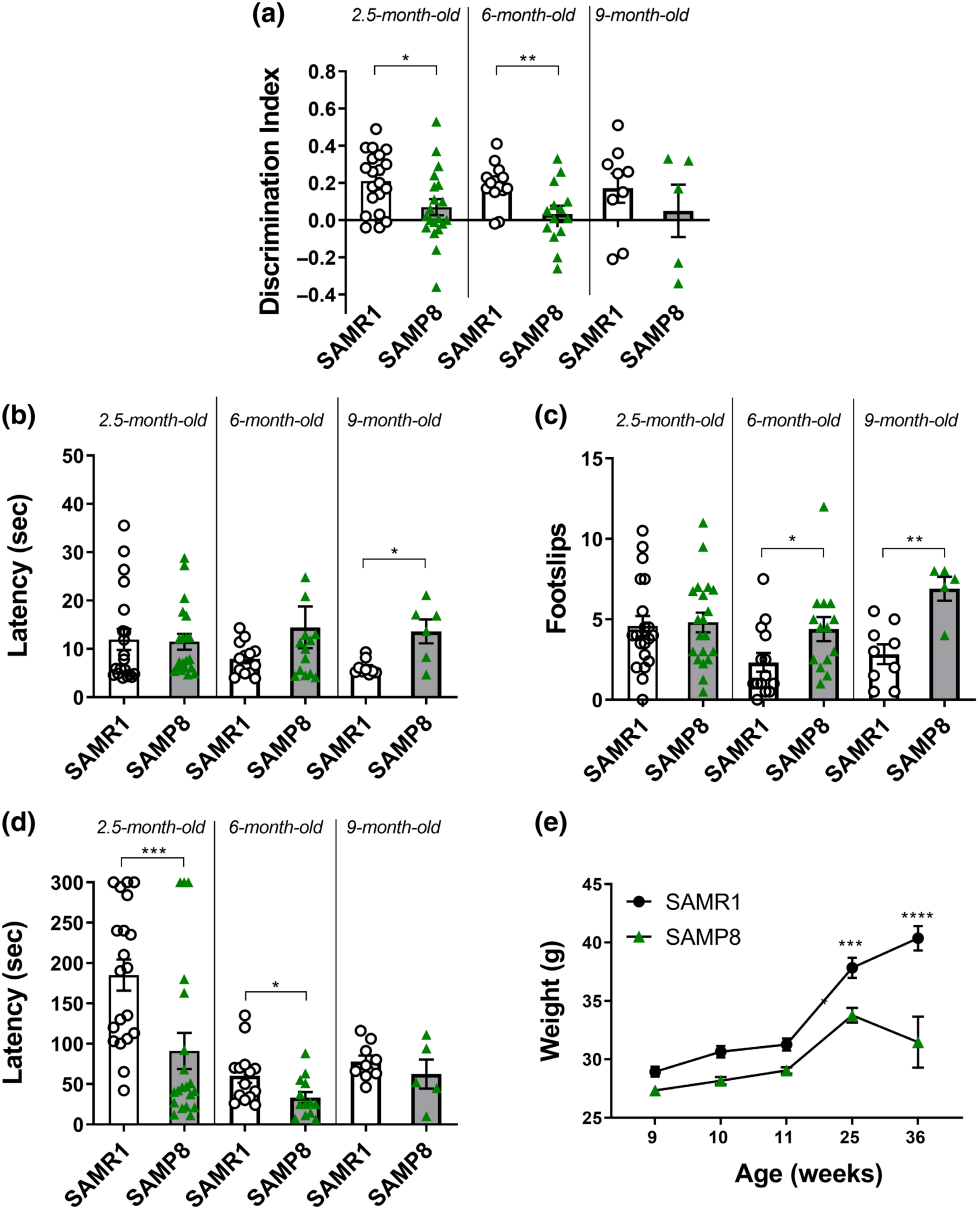
SAMP8 mice show significant memory deficits, decrease in balance and strength and weight loss throughout aging. Scatter plot with bar are mean ± standard error mean (SEM) of: (a) the discrimination index (DI) of SAMR1 and SAMP8 mice at 2.5, 6 and 9 months of age tested in the NORT (2.5 months: *t*_38_ = 2504, *p* = 0.0167; 6 months *t*_26_ = 2976 *p* = 0.0062); (b) latency (s) to cross the wooden beam (9 months: *p* = 0.0372 Mann–Whitney test) and (c) the number of foot slips for SAMR1 and SAMP8 tested in the beam walking test at 2.5, 6, and 9 months of age (6 months: *p = 0.0161; 9 months: **p = 0.0050 Mann–Whitney test); (d) mouse clinging latency on the grid in the paw grip test (2.5 months: ***p = 0.0009; 6 months: **p = 0.0187 Mann–Whitney's test). (e) Mean ± SEM of SAMR1 and SAMP8 mouse weight. Two‐way ANOVA found a significant interaction *genotype × time F*_(4,152)_ = 7664, *p* < 0.0001; ***p < 0.001; ****p < 0.0001 Tukey's multiple comparisons test.

SAMP8 mice are well known to develop cognitive deficits. Thus, in order to characterize SAMP8 mice at cognitive level throughout aging also in our lab before neuropathological and omics analyses, we tested SAMR1 and SAMP8 mice in the novel object recognition test (NORT) and Y‐maze at 2.5, 6, and 9 months of age. NORT measures long‐term recognition memory, relies on spontaneous animal behavior, and is the most widely used memory test (Balducci et al., 2015; Balducci & Forloni, 2014; Mancini et al., 2017; Vogel‐Ciernia & Wood, 2014), whereas the Y‐maze is a measure of spatial working memory. As shown in Figure 1a, SAMP8 mice show a significant impairment in recognition memory as indicated by a lower discrimination index (DI) at 2.5 and 6 months of age compared to SAMR1. No significant differences were found with the Y‐maze throughout aging, indicating no alterations in short‐term memory in SAMP8 mice.

### 2.2 Motor alterations in SAMP8 mice

Frail aging is characterized by a progressive loss of balance as well as a reduction in muscle strength and function, which can lead to the onset of sarcopenia (Clegg et al., 2013). Sarcopenia/physical frailty is nowadays emerging as risks factor for the development of frailty and seems to predict the onset of cognitive decline (Ruan et al., 2015; Wilkins et al., 2013; Yang et al., 2023). We, thus, assessed mouse balance and muscle strength in SAMP8 mice through the beam walking test and the paw grip, respectively, again from 2.5 to 9 months of age. On the beam walking test, SAMP8 mice showed a longer latency to cross the wooden beam starting at 6 months, which reached significance at 9 months, mainly because of an improving performance of SAMR1 throughout aging not achieved by SAMP8 mice (Figure 1b); the number of foot sleeps was significantly higher in SAMP8 at 6 and 9 months of age (Figure 1c), clearly indicating an impairment of their balance. In the paw grip test, SAMP8 mice showed a significantly lower clinging resistance at 2.5 and 6 months of age, thus indicating also a loss in muscle strength (Figure 1d).

### 2.3 Weight loss is a hallmark of SAMP8 mice

Considering that anorexia is a typical feature of frail subjects (Picca et al., 2022), SAMP8 and SAMR1 mouse weight was recorded throughout aging, from the ninth to the 36th week of life to assess the trend of body weight over time. Weight increased in both SAMR1 and SAMP8 mice throughout aging until week 25, when a significance weight difference between the two groups started to be appreciable. From week 25, SAMP8 mouse weight started to decline progressively compared to SAMR1, increasing the group divergence (Figure 1e).

### 2.4 Functional alterations in SAMP8 mice are associated with a defect in brain development, lower neuronal density and loss of glial cells

**FIGURE 2:**
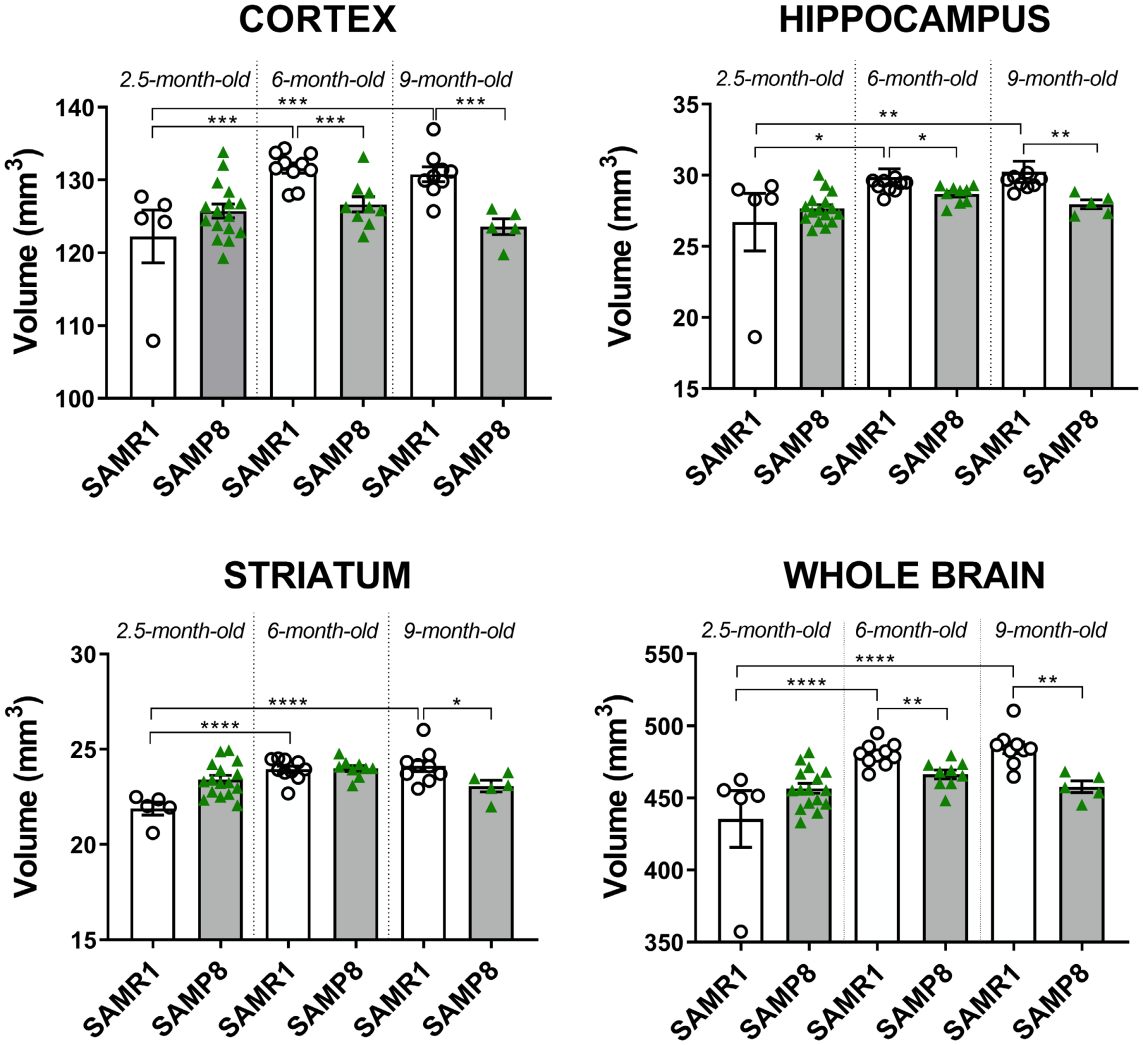
SAMP8 mouse brain shows growth defects throughout aging. Scatter plot with bar are mean ± SEM of cortical, hippocampal, striatum and whole brain volume of SAMR1 and SAMP8 at the three ages investigated. Statistical analysis carrying by comparing SAMP8 vs. SAMR1 at each single age revealed statistical significance for: Cortex (6 months) *t*_17_ = 4043, *p* = 0.0008, (9 months) *t*_12_ = 4497, *p* = 0.0007, Student's *t*‐test. Hippocampus (6 months) *p* = 0.0133, (9 months) *p* = 0.0020, Mann–Whitney's test. Striatum (9 months) *t*_12_ = 2281, *p* = 0.0416 Student's *t*‐test. Whole brain (6 months) *t*_17_ = 3583, *p* = 0.0023, (9 months) *t*_12_ = 4248, *p* = 0.0011 Student's *t*‐test. Statistical analysis through a two‐way ANOVA for factors *strain* and *time* revealed a significant interaction: *F*_(2,48)_ = 8.185, *p* = 0.0009 and a significant effect of age highlighting volume increase only for SAMR1 mice: *F*_(2,48)_ = 13.08, *p* < 0.0001. *p < 0.05, **p < 0.001, ***p < 0.001, ****p < 0.001 (Student's *t*‐test; Šidàk's test).

It is widely reported that cerebral atrophy well correlates with cognitive and motor dysfunction in frail subjects (Del Brutto et al., 2017; Gallucci et al., 2018; Kallianpur et al., 2016; Yamada et al., 2013). In order to establish whether functional deficits were associated with brain structural alterations also in our SAMP8 mice, we executed longitudinal brain MRI analysis following behavioral assessment at each age considered (2.5, 6, and 9 months). As shown in Figure 2, we found that while the cortical, hippocampal, striatal, and whole brain volume of SAMR1 mice significantly increased progressively, from 2.5 to 6 and 9 months of age, this increase did not occur in SAMP8 mice. Indeed, the SAMP8 volume was significantly lower starting at 6 months for the all areas analyzed, but for the striatum, which reached significance only at 9 months.

**FIGURE 3:**
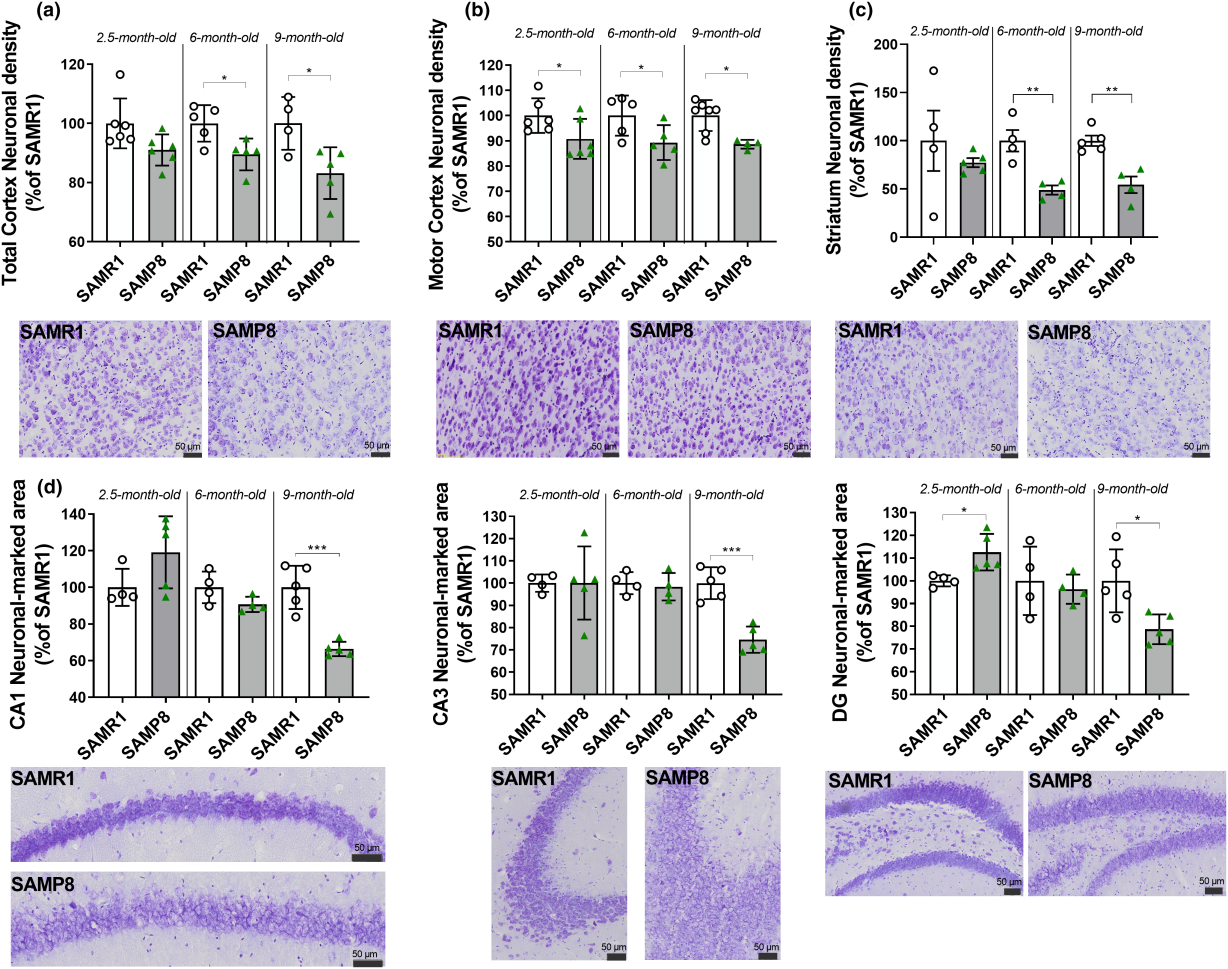
SAMP8 mice display progressive reduction in neuronal density in smaller brain areas compared to SAMR1. Scatter plot with bar are mean ± SEM of neuronal density for: (a) whole cortex (*p* = 0.0152; Mann–Whitney, 2.5 months; *t*_8_ = 2.865, *p* = 0.0210, 6 months; *t*_7_ = 2.842, *p* = 0.0250, Student's *t*‐test; 9 months); (b) motor cortex (*p* = 0.1320 Mann–Whitney, 2.5 months; *t*_8_ = 2.269, *p* = 0.0530 Student's *t*‐test, 6 months; *p* = 0.0121 Mann–Whitney, 9 months); (c) striatum (*t*_7_ = 0.8097, *p* = 0.4448, 2.5 months; *t*_6_ = 4228, *p* = 0.0055, 6 months; *t*_7_ = 4703, *p* = 0.0022 Student's *t*‐test, 9 months); (d) neuronal‐marked area for hippocampal subregions (*CA1*: *p* = 0.1905 Mann–Whitney, 2.5 months; *t*_6_ = 1.944, *p* = 0.0998, 6 months; *t*_8_ = 6.029, *p* = 0.0003, 9 months; Student's *t*‐test. *CA3*: *T*_7_ = 0.0068, *p* = 0.9948, 2.5 months; *t*_6_ = 0.4061, *p* = 0.6988, 6 months; *t*_8_ = 6.102, *p* = 0.0003, 9 months; Student's *t*‐test. *DG*: *T*_7_ = 2985, *p* = 0.0204, 2.5 months; *t*_6_ = 0.4486, *p* = 0.6694, 6 months; *t*_8_ = 3120, *p* = 0.0142, 9 months; Student's *t*‐test). Representative images of NISSL‐stained sections of the cortex, striatum and hippocampal subregions compare SAMR1 with SAMP8 mice at 9 months of age. *p < 0.05, **p < 0.001, ***p < 0.001.

Based on these results, we also addressed whether smaller brain area volumes were accompanied by lower neuronal density. To this aim, we counted neurons in the motor and whole cortex, striatum, and the neuronal‐marked area in the CA1, CA3, and dentate gyrus (DG) hippocampal subareas. As shown in Figure 3, we found that in SAMP8 mice there was a significant lower number of neurons in all the analyzed areas, which reached significance already at 2.5 months for the motor cortex, 6 months for whole cortex and striatum, and 9 months for all the investigated areas.

**FIGURE 4:**
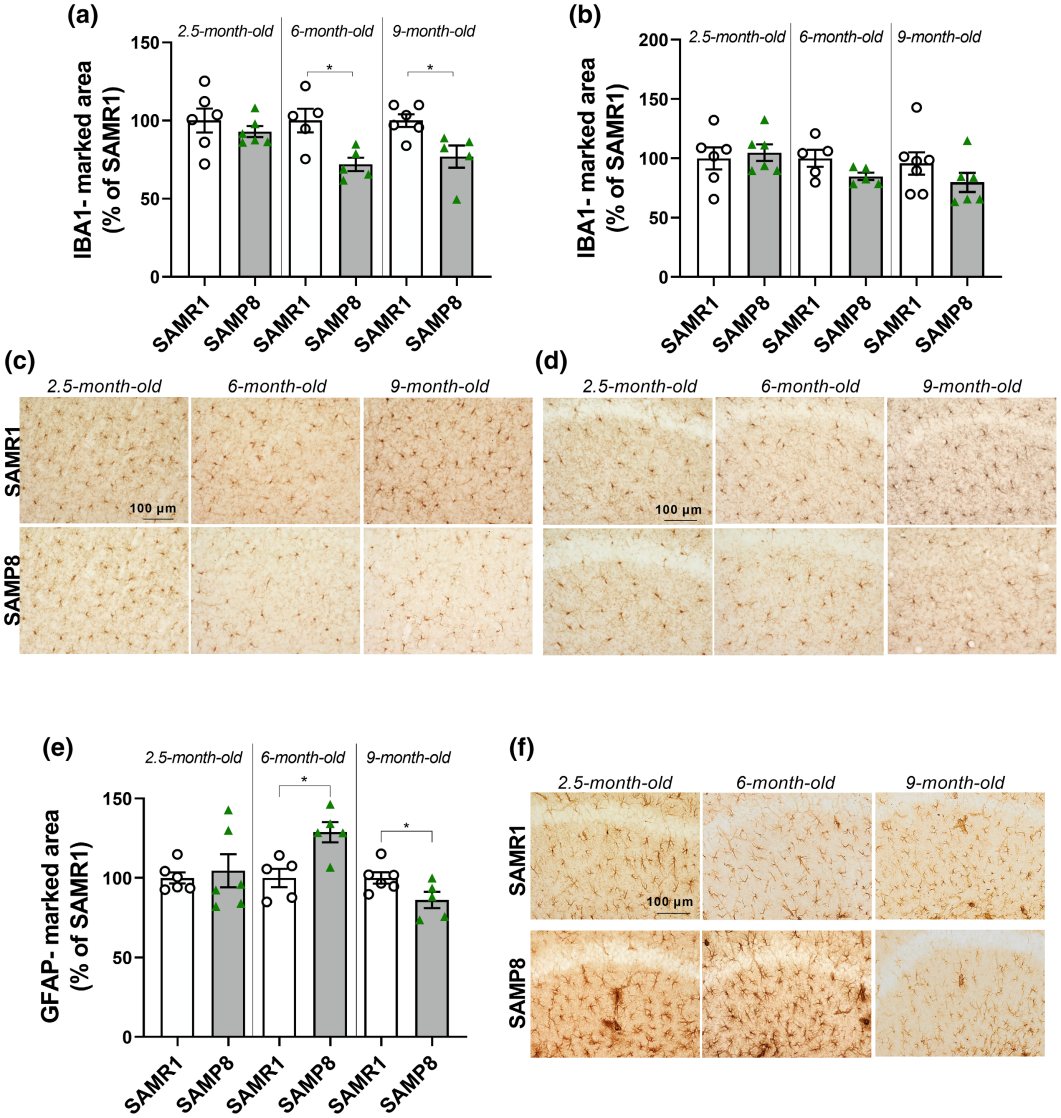
SAMP8 mice show changes in glial cell density throughout aging. Scatter plot with bar are mean ± SEM of Iba1‐marked area, immunolabelling microglial cells, in the (a) cortex (6 months: *T*_8_ = 3213, *p* = 00124; 9 months: *T*_9_ = 2947 *p* = 0.0163 Student's *t*‐test) and (b) hippocampus of SAMR1 and SAMP8 mice at 2.5, 6, and 9 months of age. (c, d) Representative images of cortical and hippocampal brain slices immunostained with the anti‐Iba1 antibody comparing cell density at the three ages investigated. (e) Scatter plot with bar are mean ± SEM of GFAP‐marked area, immunolabelling astroglial cells in the hippocampus (6 months: *T*_8_ = 3335, *p* = 0.0103; 9 months: *T*_9_ = 2287, *p* = 00480 Student's *t*‐test). (f) Representative images of hippocampal brain slices immunostained with the anti‐GFAP antibody comparing cell density at the three ages investigated in the two mouse groups; *p < 0.05.

Aging also causes profound changes in the immune system by establishing a low‐grade chronic inflammation called “inflammaging” (Fulop et al., 2023), including the activation of microglia and astrocytes in the brain, which might impact on cognitive functions (Morris et al., 2013; Ownby, 2010). We, thus, also investigated the neuroinflammatory state in SAMP8 mouse brain. In contrast to what was expected from the literature, we found no evidence of microgliosis (Edler et al., 2021). Rather, microglial cells, immunolabeled with the anti‐Iba1 antibody, showed a progressive, significant decrease in the area they occupied in the cortex (Figure 4a,c). No significant differences were found in the hippocampus, although a tendency toward reduction was detectable (Figure 4b,d). In contrast, when astrocytes were immunolabeled with the anti‐GFAP antibody, we found a significant increase in the hippocampal‐marked area at 6 months of age and a significant decrease at 9 months (Figure 4e,f).

### 2.5 Specific transcriptomic signatures distinguish frail SAMP8 from robust SAMR1 mice

**FIGURE 5:**
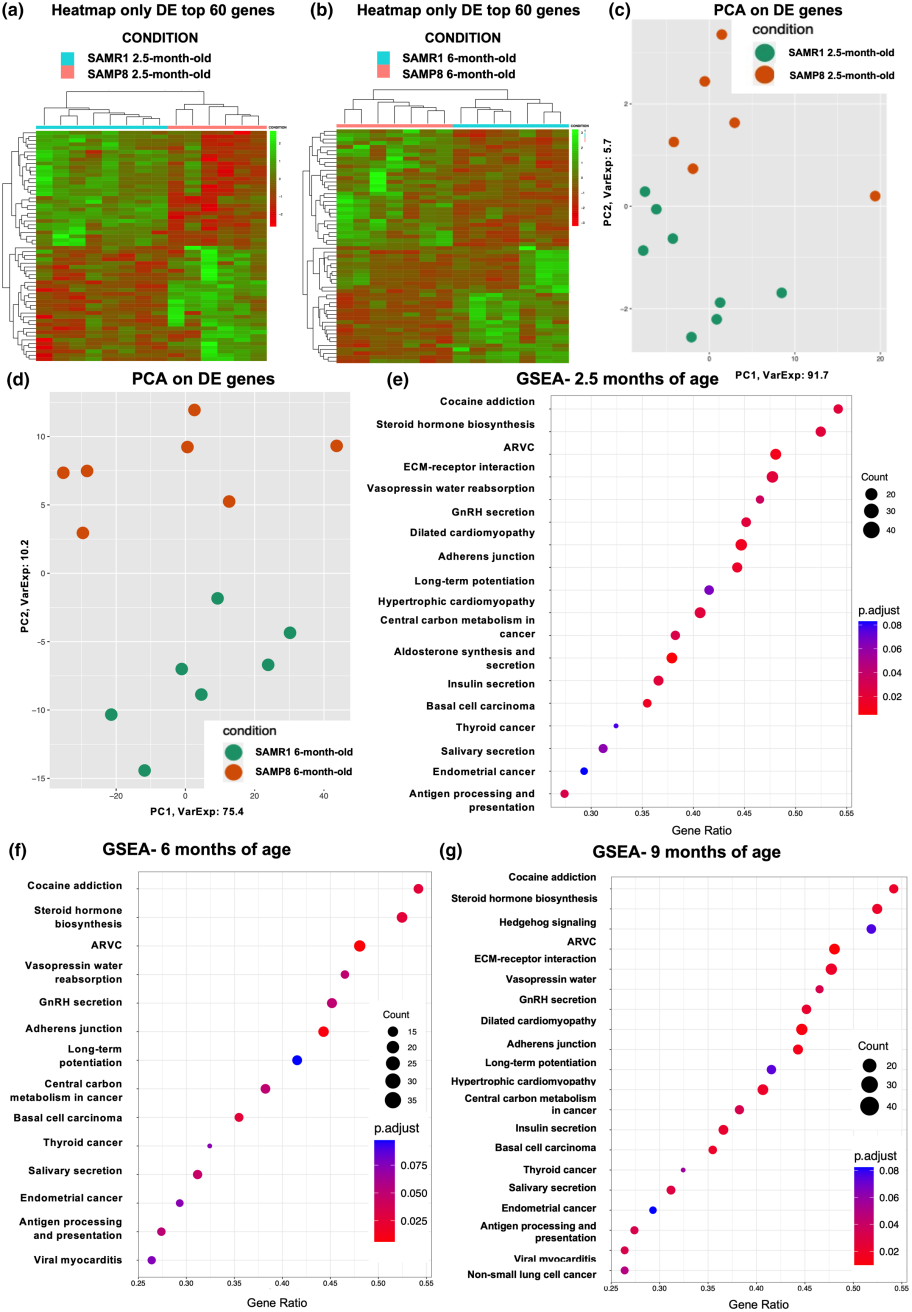
SAMP8 mice are characterized by transcriptomic signatures. Heatmaps of top 60 DE genes in SAMP8 with respect to SAMR1 mice at 2.5 (a) and 6 months (b). In red are represented downregulated DE genes, while in green upregulated ones. PCA plots of DE genes in SAMP8 with respect to SAMR1 mice at 2.5 (c) and 6 months (d). KEGG GSEA for SAMP8 compared to SAMR1 mice at 2.5 (e), 6 (f), and 9 months (g). The y‐axis represents the name of the pathway, the x‐axis represents the gene ratio, dot size represents the number of different genes, and the color indicates the adjusted *p*‐value.

In parallel to neuropathological assessment, we also carried out a transcriptomic analysis in the whole cortex and the hippocampus. These brain areas were selected based on the fact that SAMP8 mice are mainly considered as a model of sporadic AD, and we focused our attention on brain areas involved in cognitive functions. A bulk RNA‐Seq comparing the transcriptome profiles of SAMP8 mice at 2.5, 6, and 9 months of age with respect to their matched SAMR1 controls was performed. Cortex and hippocampus were considered as covariates in our statistical model since they could influence gene expression differently. Genes with |log_2_(SAMP8/SAMR1)| ≥ 1 and false discovery rate (FDR) ≤ 0.1 were considered as differentially expressed (DE) and were retained for further analysis. Heatmap and principal component analysis (PCA) of the DE RNAs in SAMP8 mice at 2.5 and 6 months of age showed a clear separation between SAMP8 and SAMR1, highlighting the presence of different expression profiles and suggesting that frailty may have an important impact on many cellular features (Figure 5a–d). A total deregulation of 8 DE genes (5 out of 8 resulted upregulated and 3 out of 8 resulted downregulated) was found in SAMP8 versus SAMR1 at 2.5 months of age (Figure S1A, Table 1 and Table S1). Interestingly, in SAMP8 at 6 months of age, we detect many differences in gene expression profiles compared to SAMR1, since 146 genes emerged as deregulated. Specifically, 95 out of 146 were upregulated while 51 out of 146 were downregulated (Figure S1B, Table 1 and Table S2). Moreover, no significantly deregulated genes were found when considering SAMP8 with respect to SAMR1 at 9 months of age (Table 1).

**TABLE 1:** The differential expression analysis highlighted a dysregulation in transcriptomic profiles in SAMP8 mice at 2.5, 6 and 9 months of age with respect to their matched controls.

	Upregulated	Downregulated	Total
SAMP8 vs. SAMR1 at 2.5 months of age	5	3	8
SAMP8 vs. SAMR1 at 6 months of age	95	51	146
SAMP8 vs. SAMR1 at 9 months of age	–	–	–

To further investigate the differences between SAMP8 mice and their respective controls at different months of age, we performed and extensive Gene Set Enrichment Analysis (GSEA) on Kyoto Encyclopedia of Genes and Genomes (KEGG). Frailty implicates changes in gene expression that affects pathways related to metabolism (Zhang et al., 2020). Indeed, the GSEA KEGG analysis highlighted 18 deregulated pathways when considering SAMP8 mice at 2.5 months of age with respect to SAMR1 at the same age (Figure 5e). Interestingly, all pathways were found to be downregulated (Table S3). This downregulation pattern persisted in SAMP8 mice at 6 and 9 months of age when compared to their respective control groups. Despite the fact that at 9 months we did not find significantly deregulated genes in SAMP8 mice, GSEA still highlighted some altered pathways, which is justified by the fact that GSEA considers the whole transcriptome.

Specifically, GSEA KEGG pathways analysis highlighted 14 and 20 deregulated pathways (all downregulated), respectively (Figure 5f,g and Tables S4 and S5). Furthermore, our GSEA results demonstrated a consistent downregulation of metabolic pathways across all comparisons, including pathways associated with “Cocaine addiction” and “Steroid hormone biosynthesis” (Figure 5e–g).

### 2.6 Plasma metabolomics highlights lipid changes in SAMP8 mice already at 2.5 months of age

**FIGURE 6:**
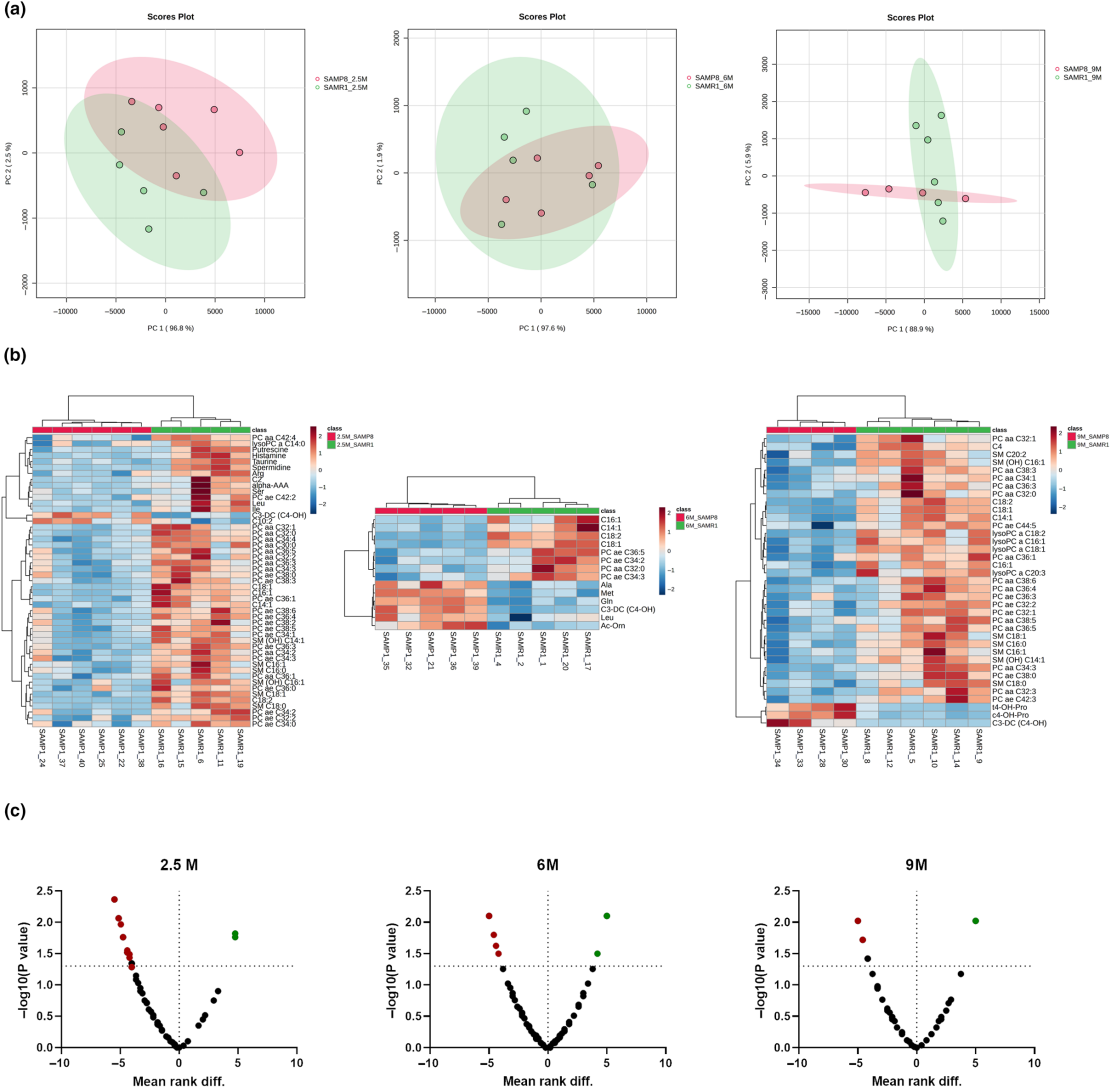
Plasma metabolomics changes in SAMP8 mice are observed already at 2.5 months of age. OPLS‐DA score plots representative of the metabolomics differences between SAMP8 and SAMR1 at 2.5, 6, and 9 months of age respectively (a). Volcano plots represent the metabolic statistically significant difference (Wilcoxon–Mann–Whitney) between SAMP8 and SAMR1 at 2.5, 6, and 9 months of age respectively. Red and green dots highlighted the increased and decreased statistically significant different metabolites in the comparison of interest (b). Hierarchical clustering heatmaps of the statistically significant different metabolites between SAMP8 and SAMR1 at 2.5, 6, and 9 months of age, respectively (c).

At a systemic level, we evaluated temporal metabolic alterations between SAMP8 and SAMR1 mice in the plasma. Targeted metabolomics strategy quantified a total of 147 metabolites, 13 biogenic amines, one sugar, 21 amino acids, 10 acylcarnitines (ACs), 13 lysophosphatidylcholines (lysoPCs), 74 phosphatidylcholine (PCs), and 15 sphingomyelins (Table S6). Metabolic states were assessed at 2.5, 6, and 9 months of age using multivariate and univariate approaches. Principal component analysis showed alterations in the target plasma metabolomics profiles at 2.5 months, progressively decreasing at 6 and 9 months (Figure 6a). These differences were also confirmed by the univariate analysis (Wilcoxon–Mann–Whitney) that showed significant levels alteration of 49, 14, and 37 metabolites between SAMP8 and SAMR1 at 2.5, 6, and 9 months, respectively (Table S7). Focusing on the significantly altered metabolites, we observed that SAMP8 mice were characterized by generally low plasma levels of ACs and lipid species compared to SAMR1 mice (Figure 6b,c and Table S6). This metabolic asset was maintained to a lesser extent at 6 and 9 months (Figure 6b,c and Table S7).

To identify possible links between plasma metabolic alterations and frailty‐related indicators, we performed a correlation analysis between statistically significant different plasma metabolites and their pathological outcomes at 2.5, 6, and 9 months of age. We observed major significantly positive correlation pairs (spearman correlation *p* < 0.05, *r* ± 0.8) between metabolites and various neuropathology indicators at all ages investigated. Whereas, cognition and brain structural parameters are well correlated with metabolite changes only at 2.5 and 9 months of age, respectively (Table S8A–C). Notably, the correlation pairs involved mainly ACs and lipid species.

### 2.7 Integrated pathways analysis reveals age‐associated molecular signatures in SAMP8 mice

Lastly, we employed the MetaboAnalyst 6.0 webtool to integrate transcriptomic and metabolomic data, aiming to elucidate the molecular mechanisms underlying the aging process in SAMP8 mice. Specifically, we conducted an integrative pathways analysis for both 2.5‐month‐ and 6‐month‐old SAMP8 mice compared to age‐matched SAMR1 controls. By this approach, we aimed to capture comprehensive insights into the dynamic changes occurring with aging. This comprehensive analysis revealed a total of 40 pathways at 2.5 months and 165 pathways at 6 months of age, with details provided in the Table S9. Among these, 23 pathways were found to be shared between the two age groups, as shown by the Venn diagram in Figure S1C. Notably, shared pathways include those associated with “mTOR signaling,” “GABAergic synapse,” “Long‐term potentiation,” and “Glutathione metabolism,” indicative of the intricate interplay between synaptic functions, neuronal plasticity, and metabolic regulation during aging (Table 2). This approach allowed us to pinpoint common biological processes and pathways affected during the aging process in SAMP8 mice and linked to our functional deficits, providing valuable insights into the molecular mechanisms underlying age‐related changes in this model organism.

**TABLE 2:** Shared pathways identified through integrative pathways analysis comparing 2.5‐month and 6‐month‐old SAMP8 mice with age‐matched SAMR1 controls.

Number of shared pathways	23
SAMP8 vs. SAMR1 2.5 months old and SAMP8 versus SAMR1 6 months old	mTOR signalling pathway
	Mineral absorption
	Gastric acid secretion
	Cysteine and methionine metabolism
	Aminoacyl‐tRNA biosynthesis
	Valine, leucine, and isoleucine biosynthesis
	Inflammatory mediator regulation of TRP channels
	Salmonella infection
	Neuroactive ligand–receptor interaction
	Insulin resistance
	Central carbon metabolism in cancer
	ABC transporters
	GABAergic synapse
	Arginine biosynthesis
	Lysine degradation
	Synaptic vesicle cycle
	Long‐term potentiation
	Bile secretion
	Protein digestion and absorption
	Adrenergic signalling in cardiomyocytes
	Glutathione metabolism
	Valine, leucine, and isoleucine degradation
	Chagas disease (American trypanosomiasis)

## 3 DISCUSSION

Aging is the main risk factor for the development of a frail condition leading to several pathologies including neurodegenerative diseases, of which AD represents the most diffused form of dementia. The main diagnostic tools are the *Fried frailty phenotype*, a measure of physical performances (Fried et al., 2001), and the *Frailty Index*, which is instead based on the comparison of multiple and cumulative deficits and was created by Rockwood and colleagues (Clegg et al., 2013). However, these indices have the limit of detecting frailty only when it has reached a level of severity that makes the deleterious effects evident. Moreover, they can be affected by the subjectivity of individual answers and lack of linkage with the biological mechanisms of frailty (Whitson et al., 2007).

The identification of early biomarkers predicting frailty would, indeed, represent a breakthrough for the clinic since it will allow to identify the related biomolecular mechanisms and develop precision medicine‐based approaches so to prevent frailty and guarantee a healthier aging. This will have an impact at global level, also considering the foreseen increase in life expectancy.

We herein described a longitudinal characterization at functional (motor and cognitive abilities) and brain structural (MRI) level of SAMP8 mice, compared with their respective normally aging control SAMR1, combined with brain transcriptomic and plasma metabolomic analyses, aimed at identifying predictive biomarkers of frailty. SAMP8 are widely used as a senescence mouse model spontaneously developing a frail phenotype converting to AD‐like dementia (Liu, Chang et al., 2020).

At functional level, we found that SAMP8 mice displayed, already at 2.5 months of age, a significant impairment in long‐term recognition. At motor level, they showed a significant reduction in paw grip strength at 2.5 and 6 months, as well as balance deficits starting at 6 months. Of note, recent studies have shown that older adults with accelerated declines in gait and memory have higher risk of developing dementia than those with either solely memory or gait decline (Montero‐Odasso et al., 2018; Tian et al., 2020). This evidence further promotes SAMP8 mice as the ideal model to find predictive biomarkers of aging‐related dementia (Folch et al., 2018; Mori & Higuchi, 2019).

SAMP8 functional alterations were associated with a reduction in brain volume growth, indeed, whereas in SAMR1 mice there was a significant volume increase of the whole brain and of specific areas such as the whole cortex, hippocampus, and striatum throughout aging, in SAMP8 mice, this volume growth did not occur, and by the age of 6 months the volume differences between SAMR1 and SAMP8 became significant. We hypothesize that the senescent condition of SAMP8 might imply a neurodevelopment defect. We have no precise explanation for this defect, which will be further addressed with future studies, but it is conceivable to assume that it might be the result of a congenital condition combined with aging‐related effects. To be noticed that few studies on neurogenesis in SAMP8 mice proved accelerated depletion of the hippocampal neuronal stem cell (NSC) pool compared with SAMR1 around 6 months of age or even later, coinciding in time and space with a reduction in neurogenesis and an increase in astroglial differentiation, although the latter is not always confirmed (Díaz‐Moreno et al., 2013; Gang et al., 2011; Gómez‐Oliva et al., 2023; Pačesová et al., 2022); no defects were observed at early ages. In addition, a significant increase in immature neurons and a significant decrease in mature neurons was found at 5 months, which worsen at 10 months of age (Gang et al., 2011). Notably, age‐related drifts in histone posttranslational modifications in the brain of SAMP8 mice were described as possible responsible for neurodevelopment defects. These age‐related drifts in brain epigenomes would negatively affect neuronal transcriptomes (Fischer et al., 2010; Lu et al., 2004), contributing to a signaling capacity decline of neurons, defects in axon myelination, and other molecular alterations that have been linked to various cognitive disorders and eventually neurodegeneration (Jakovcevski & Akbarian, 2012; Yankner et al., 2008).

These data fully agree with our findings; indeed, in addition to brain volume differences, we also found that the neuronal density in the same smaller SAMP8 brain areas became progressively lower. These brain areas are mainly involved in the regulation of cognitive and motor functions. Indeed, we found significant neuronal density reduction in the motor cortex and striatum associable with motor impairment and in the whole cortex and hippocampus well known to be involved in learning and memory processing (Bettio et al., 2017; Kreitzer, 2009; Vitrac & Benoit‐Marand, 2017). Beside a progressive decrease in neuronal density, we found that also the area occupied by microglial cells decreased throughout aging in SAMP8 mice, mostly in the cortex, although a tendency was observed also in the hippocampus. These findings contrast with previous published data showing microgliosis in the cortex of SAMP8 mice at 6 months and in the brain stem at 9 months (Pačesová et al., 2022), discrepancies that require further elucidation, considering that this increase is not even detected in the same brain areas among research groups.

In contrast, the marked area occupied by astrocytes significantly increased at 6 months in the hippocampus, indicating the presence of astrogliosis at a certain age, which agrees with the previous studies mentioned above (Gómez‐Oliva et al., 2023), describing an increased astrogliosis at 6 months in association with a reduction in neurogenesis. However, as for microglia, at 9 months also the marked area occupied by astrocytes decreased in our SAMP8 mice, which is in line with data from Gang et al. (2011). We have no explanation for glial cell reduction yet, but future dedicated studies will be devoted to better characterize their morphology/reaction and phenotype so to clarify these changes. However, we cannot exclude that SAMP8‐related epigenetic changes described above for neurons might also influence the survival of glial cells throughout aging in a frail context (Fischer et al., 2010; Lu et al., 2004). It is known, for instance, that a multitude of factors, including cellular aging, transcriptomic changes, senescence‐associated secretory phenotype, dysregulated glial metabolism, and the milieu in which these cells reside, could influence their behavior and survival (Minhas et al., 2021; Palmer & Ousman, 2018; Van den Bossche & Leenen, 2021). A recent paper reported that SAMP8 mice treated with LPS showed, and immune microglial response at 6 months but null responsiveness at 12 months of age, likely suggesting an exhaustion of their activity or immune suppression throughout SAMP8 frail aging (Molina‐Martínez et al., 2020).

The possibility to identify predictive biomarkers well relating to functional deficits by exploiting omics‐based approaches helps in exploring the physiological mechanisms underlying frailty and aids in evaluating the frailty risk development and progression. Indeed, complementary omics analyses on mouse brains and plasma allowed us to identify early metabolomic and transcriptional changes capable of distinguishing frail SAMP8 from robust SAMR1 mice even at young ages.

Through transcriptomics analysis of the cortex and the hippocampus, we have identified a series of deregulated genes mainly distinguishing SAMR1 from SAMP8 at young ages, 2.5 and 6 months. The higher number of deregulated genes was found at 6 months. Surprisingly, no significant differences were found at 9 months when the genetic profile turned out to be more aligned between the two strains, thus indicating that the more reliable biomarkers are detectable at earlier stage of frailty development. Of note, two main pathways emerged as significantly altered, which were related to cocaine addiction and steroid hormone biosynthesis.

Interestingly, the reward system (i.e., cocaine addiction) has been tightly linked to motor and cognitive deficits (Lauretani et al., 2022). Patients with motoric cognitive risk (MCR) syndrome, for instance, show a smaller volume of total gray matter, cortical gray matter, motor cortex, prefrontal cortex, and dorsolateral segment of the prefrontal cortex, when compared to those who do not meet the diagnostic criteria for MCR (Beauchet et al., 2016). These brain areas compose functional circuits and neuronal networks shared by the motor, cognitive, and reward systems, which includes the mesolimbic pathway, some nuclei of the terminal stria, the amygdala, and the hippocampus. The preservation of these integrated systems is fundamental in the elderly patient for resilience to cognitive and motor deficits (Lauretani et al., 2022). Based on this evidence, and considering that in our SAMP8 mice at 6 months we observe both motor impairment and cognitive dysfunction, as well as smaller volume of total gray matter, motor and whole cortex and hippocampus followed by a striatal reduction at later ages, it is conceivable to assume that this transcriptomic signature is highlighting a major compromising of the reward system networking likely contributing to cognitive and motor deficits.

The other downregulated pathway found in SAMP8 mice at 6 months is that related to steroid hormone biosynthesis. Steroid hormones are synthesized by the gonads and adrenal glands and easily cross the BBB, but they are also synthesized by neurons and glia in the central nervous system (CNS) (Baulieu et al., 2001; Schumacher et al., 2003). It is well known that steroids and neurosteroids have pleiotropic effects in the CNS: They are essential for neuronal viability, maintenance, and repair of myelin sheaths and are involved in the regulation of synaptic plasticity, dendritic spine development, learning, and memory (Raciti et al., 2023). Thus, downregulation of this pathway might predict lower levels of steroids, which could be related to cognitive dysfunction and neuronal loss found in SAMP8 mice. The reduction in circulating steroid hormones is, indeed, associated with several age‐related pathologies that may influence human health span, whereas treatment with steroid hormones in mice has shown protective effects in terms of mitochondrial activity, neuronal viability, and cognitive functions (Schumacher et al., 2003; Velarde, 2014). Mitochondria play major roles in the biosynthesis of sex steroid hormones, which, in turn, regulate mitochondrial activity; notably, SAMP8 mice have mitochondrial alterations and increased oxidative stress (Brunetti et al., 2020; Liu, Chang et al., 2020). To date, it remains elusive whether changes in the synthesis of neurosteroids at brain level throughout aging play a role in the aging process. For instance, controversial data in women relate the reduction of estrogen with the presence of AD, with a positive correlation found in some cases and no differences in some others (Cunningham et al., 2001; Manly et al., 2000; Schönknecht et al., 2001; Wong et al., 2023). However, our data seem to confirm that in a congenital frail condition, alteration in the biosynthesis of steroid hormones emerges as mainly downregulated pathways.

Through, in parallel, plasma metabolomics analysis, we found changes in 147 metabolites, but the main differences between SAMR1 and SAMP8 mice, highlighted by PCA analysis, occurred at 2.5 months of age and progressively diminished at 6 and 9 months. Particularly, the plasma metabolites significantly lower in SAMP8 mice were ACs and lipid species such as lysoPCs, PCs, and sphingomyelin, compared to SAMR1 mice.

ACs are a class of metabolites generated in the mitochondria (McCann et al., 2021) predictive of multiple geriatric syndromes such as frailty and disorders affecting the musculature (Ng et al., 2022). During normal physiology, ACs facilitate the transport of fatty acids into mitochondria and are essential for β‐oxidation and energy metabolism (Knottnerus et al., 2018), but they also restore cell membranes and synaptic function, enhance cholinergic activity, protect from toxins, and exert neurotrophic actions (Pennisi et al., 2020). In primary carnitine deficiency, the level of plasma ACs is low due to impaired formation, and muscle weakness and cardiomyopathy are typical consequences. Accordingly, SAMP8 mice, beside weakness and motor and cognitive deficits, are also characterized by aging‐related miocardial alterations (Karuppagounder et al., 2017).

We found significant constitutively lower levels of ACs in our SAMP8 mice already at 2.5 months of age compared to SAMR1, which agrees with a previous study conducted on robust, pre‐frail, and frail subjects showing a progressive AC lowering in the plasma of pre‐frail and frail subjects (Malaguarnera et al., 2020) and recalls our transcriptomic data highlighting the steroid hormone as a main downregulated pathway and strongly implicated in the regulation of mitochondrial activity.

Of note, pre‐frail subjects receiving a 3‐month treatment with acetyl‐l‐carnitine showed an increase in serum‐free carnitine and ACs, associated with an improvement in Mini‐Mental State Examination and in 6‐walking distance test (Malaguarnera et al., 2022). Several works encourage to consider ACs in the therapy of frailty, dementia, and neurodegenerative diseases, considering that starting from 1991, when positive effects were already described in AD patients (Spagnoli et al., 1991), promising evidence continues to emerge (De Marchi et al., 2023; Maldonado et al., 2020; Pennisi et al., 2020). We, thus, herein speculate that the constitutively ACs deficiency in our SAMP8 mice might have predisposed to the establishment of cellular dysfunctions leading to the development of cognitive and motor deficiency, and their lower levels might be considered as predictive biomarkers of frailty.

LysoPC was the other metabolite we found reduced in the plasma of SAMP8 mice. LysoPCs are a major class of glycerophospholipids in human blood, relevant to muscle mass and guaranteeing mitochondrial oxidative capacity in skeletal muscle (Gonzalez‐Freire et al., 2019; Semba et al., 2019). A decrease in blood of LysoPCs 18:2 level, for instance, has been associated with motor alterations but also with cognitive impairment; indeed, the circulating LysoPC content has been used to predict a decline in gait speed or cognition (Gonzalez‐Freire et al., 2019; Meng et al., 2022; Semba et al., 2019). LysoPCs play also important roles in inflammation and antioxidation; thus, their decrease in the circulation might permit the onset of inflammatory events and oxidative stress at peripheral and central levels widely recognized as responsible for cell suffering and cognitive impairment, both found in our mice. Tian et al. conducted a metabolomic analysis in the plasma of subjects enrolled in the Baltimore Longitudinal Study of Aging, a prospective study with continuous enrollment since 1958. Follow‐up visits occurred every 4 years for participants aged <60, every 2 years for ages 60–79, and annually for ages 80 and older in memory and gait. Authors found that metabolomic signatures of dual decline in memory and gait, a phenotype that has been previously associated with high risk of dementia, mainly included a reduction in the level of plasma LysoPCs (Tian et al., 2019, 2020, 2023). Accordingly, in our SAMP8 mice LysoPCs plasma reduction came in association with the presence of both gait and memory defects as well as neuronal/glial cell loss.

Also, changes in sphingolipid metabolism were reported as in our SAMP8 mice. Marron et al. (2019) identified metabolites and biological pathways associated with frailty in 287 black men aged 70–81 years, among which emerged sphingomyelin. Sphingolipids are pivotal constituents of the plasma membranes, highly enriched in the nervous system and essential for proper brain development and functions. Perturbation of their metabolism has been associated with development of neurological diseases (Olsen & Færgeman, 2017).

The PCs plasma reduction in SAMP8 mice might also alert on brain cell damages and suffering. PCs are phospholipids that, as for ACs, play a major role in the preservation of cell membrane integrity and function (van Meer & de Kroon, 2011). Studies have shown a decrease in plasma level of PCs predicting mild cognitive impairment or AD (Mapstone et al., 2014; Schaefer et al., 2006). Constitutive PCs plasma reduction in our frail mice, showing cognitive deficits, support these findings and might predict neuronal and glial suffering culminating in cell reduction and loss detected in our mice. Of note, in accordance with all this evidence, our Spearman correlation analysis did confirm that metabolomic changes found in SAMP8 mice well correlate with the occurrence of cognitive deficits, as well as of brain atrophy and neuropathological changes, in most cases in accordance with the degree of their severity. In contrast, no correlations were found with transcriptomic changes. We speculate that this could be due to two possible reasons: first, the limited number of samples used in the analysis, which may have affected the statistical power to detect significant associations. Second, the complexity of the relationship between gene expression and dementia indicators, considering the potential influence of post‐translational modifications and other regulatory mechanisms beyond transcriptional regulation. With reference to others regulatory mechanisms particular attention might by ascribed to epigenetic regulation of gene expression such as methylation/demethylation of DNA and histones. Studies on this topic in SAMP8 mice have been reviewed in Griñán‐Ferré et al. (2018), where they bring to light the possible participation of epigenetic modulation in the aging and neurodegenerative processes characterizing these mice.

In addition to the correlative analysis, we also carried out the integration of transcriptomic and metabolomic data, which allowed for a deeper investigation into the molecular mechanisms driving aging in SAMP8 mice. Through comparative analysis between different age groups of SAMP8 mice and age‐matched SAMR1 controls, pathways associated with critical processes such as “GABAergic synapse,” “long‐term potentiation,” and “glutathione metabolism” were identified at 2.5 and 6 months of age, highlighting connections with synaptic function, neuronal plasticity, and metabolic regulation during the aging process (Colavitta & Barrantes, 2023; Rivera et al., 2023; Wang et al., 2014) and which might explain SAMP8 mouse alterations and deficits.

In other studies where SAMP8 and SAMR1 mice were compared at different ages, some differences were found at the level of gene expression. Specifically, Fujuwara and coworkers, by comparing the two mouse strains at different time points (4 vs. 12 months), found alterations in gene expression related to many brain functions, including neurogenesis, neurometabolism, neuroinflammation, and synapse dynamics (Fujiwara et al., 2023). Accordingly, by considering the integration between gene expression and metabolomic, we also identified alterations in some of these pathways at earlier ages as described above.

Furthermore, a comprehensive multi‐omics approach has been previously applied in SAMP8 mice to investigate the relationship between aging and dementia. Currais and coworkers, by performing whole transcriptome analysis in SAMP8 animals at both 3 versus 10 months of age, revealed alterations in a number of important signaling pathways related to brain functions, including axonal guidance, G‐protein coupled receptor (GPCR), protein kinase A (PKA), cAMP, and neuronal cAMP response element‐binding protein (CREB) signaling. As mentioned above, our gene expression, as well as metabolomic evidence, even if obtained at different time points, confirmed and integrated these previously published findings (Currais et al., 2015). Interplay between epigenetic mechanisms and gene networks assumed to be relevant for a pathological aging has been described by Cosin‐Tomas and coworkers, who found dysregulated genes at 2, but—in contrast to our findings—also at 9 months of age. The differences in observations could be ascribed to a different approach applied for investigations. Specifically, these authors performed microRNA expression arrays associated with real‐time PCR. Moreover, this study has been performed in female mice (Cosín‐Tomás et al., 2018). Further studies in larger cohorts of mice will allow to further support all these findings.

In conclusion, the metabolic and transcriptomic signatures found in SAMP8 mice seem to well distinguish a frail condition from a robust one and support recent clinical studies in pre‐frail, frail, and AD subjects in need of further verification. In addition, the fact that metabolomic signatures were constitutive in SAMP8 mice—spontaneously developing a frail aging converting to AD dementia—is compelling since it indicates that these metabolite lower levels, well correlating with dementia‐related indicators, together with transcriptomic changes, could predispose to frailty development and could be considered as valuable predictive biomarkers.

## 4 EXPERIMENTAL PROCEDURES

**SCHEME 1:**
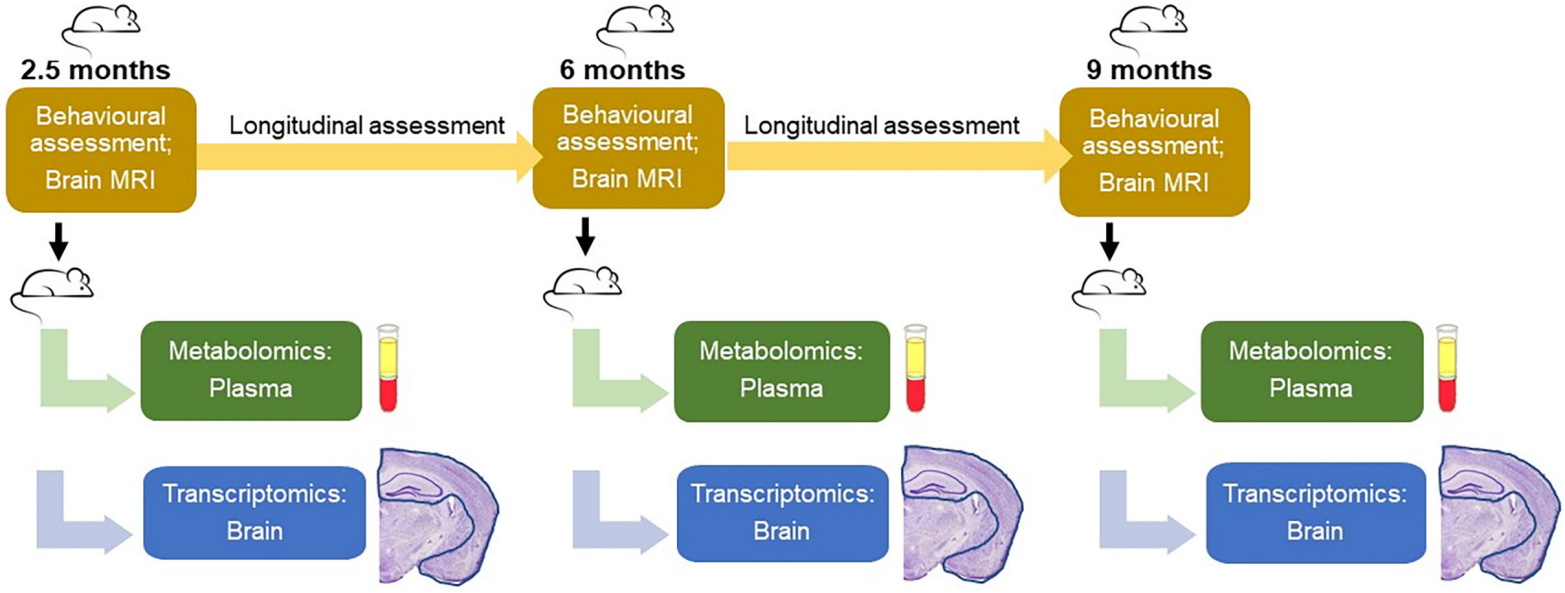
Experimental flowchart.

Mice were followed longitudinally from 2.5 to 9 months of age for in vivo analyses. At each intermediate time point of investigation, 5–6 mice were sacrificed for ex vivo analyses. Scheme 1 describes the experimental flowchart.

### 4.1 Animals

Twenty SAMR1 and 20 SAMP8 male mice were purchased by ENVIGO (Italy). Mice were all drug and behavioral test naïve, and the experiments were all conducted during the light cycle. All animals were housed in a SPF facility four/cage in standard mouse cages containing sawdust with food (2018S Envigo diet) and water ad libitum, under conventional laboratory conditions (room temperature: 20 ± 2°C; humidity: 60%) and a 12/12‐h light/dark cycle (7:00 AM–7:00 PM). The IRFMN adheres to the principles set out in the following laws, regulation, and policies governing the Care and Use of Laboratory Animals: Italian Governing Law (D.lgs 26/2014; Authorization n.19/2008‐A issued March 6, 2008 by Ministry of Health); Mario Negri Institutional Regulations and Policies providing internal authorization for persons conducting animal experiments (Quality Management System Certificate – UNI EN ISO 9001:2015 – Reg. No. 6121); the NIH Guide for the Care and Use of Laboratory Animals (2011 edition); and EU directives and guidelines (EEC Council Directive 2010/63/UE). The statement of Compliance (Assurance) with the Public Health Service (PHS) Policy on Human Care and Use of Laboratory Animals has been reviewed (9/9/2014; Animal Welfare Assurance #A5023‐01). All animals were managed in accordance with European directive 2010/63/UE and with Italian law D.l. 26/2014. The procedures were approved by the local animal health and ethical committee and were authorized by the national authority (Istituto Superiore di Sanità; authorization no. 293/2019‐PR). All efforts were made to reduce the number of animals by following the 3R's rule.

### 4.2 Novel object recognition test

Mice were tested in their home cage (30 × 15 cm) to reduce stress related to the exposure to a new environment. The following objects were used: a black plastic cylinder (4 × 5 cm), a glass vial with a white cup filled with water (3 × 6 cm), and a metal cube (3 × 5 cm). The task started with a 10 min familiarization trial during which exploration was recorded by an investigator blinded to the strain. Sniffing, touching, and stretching the head toward the object at a distance not more than 2 cm were scored as object investigation. Twenty‐four hours later (test trial), mice were exposed for 10 min to two objects: one familiar and a new, different one (novel object), and the time spent exploring the objects was recorded. Memory was expressed as a discrimination index, that is, (seconds on novel − seconds on familiar)/(tot time on objects).

### 4.3 Y‐maze

Working memory was measured with the Y‐maze. The apparatus arm was 19.5 cm long and 7.5 cm wide, with the wall of 12 cm high. Each mouse was placed in the center of the maze. The number of entries into the arms and alterations were recorded for 8 min by an operator blind to strain. The percentage of investigation was calculated as number of correct alterations/numbers of total arm entries (Kraeuter et al., 2019).

### 4.4 Beam walking test

The beam walking test measures the foot slips and latency of a mouse walking twice along an elevated wooden beam (8 mm wide and 100 cm long). Before the test, mice are trained in three habituation trials (intertrial interval: 30 s). Foot slips and latency were assessed 60 s after the last training trial. Results are expressed as mean ± SEM of the two trials (intertrial interval: 60 s) in the test phase. Mice with gait instability had more foot slips, and a longer latency than not impaired animals (La Vitola et al., 2021).

### 4.5 Paw grip test

The paw grip test has been performed as previously described (Nardo et al., 2018). Briefly, mice were placed on a horizontal grid at about 30 cm from the table, and the tail is gently pulled until they grasp the grid with their fore and hind paws. The lid is then gently turned upside down, and the latency time of the mouse to fall on the table is recorded for a maximum of 300 s. Each mouse is given up to three attempts, and the longest latency is recorded.

### 4.6 MRI

Animals were anesthetized with isoflurane in a mixture of O_2_ (30%) and N_2_O (70%). Body temperature was maintained at approximately 37°C by a warm water‐circulated heating cradle. Imaging was performed on a 7 T small bore animal Scanner (Bruker Biospec, Ettlingen, Germany) equipped with a quadrature 1H CryoProbeTM (Bruker, Ettlingen, Germany) surface coil as transmitter and receiver. A 3D fast low‐angle shot magnetic resonance imaging (FLASH) sequence has been performed to assess anatomic changes. The morphologic images were obtained with the following parameters: TR/TE 250/3 ms; flip angle 15°; image resolution 100 × 100 × 100 μm^3^; FOV 3 × 0.8 × 1.1 cm^2^; acquisition matrix 300 × 80 × 110.

### 4.7 MRI volumetric measurements

After the creation of an in‐house set of mouse brain atlases, all the anatomical brain images acquired in the study were preprocessed applying a nonuniform bias field correction (N4BiasFieldCorrection). A multi‐atlas segmentation approach (Nie & Shen, 2013) was then adopted, using the antsJointLabelFusion.sh script embedded in the ANTs software library, for brain extraction so to subsequently divide the different brain regions as defined by a mouse brain atlas.

### 4.8 Intracardiac perfusion and brain tissue preparation

Animals were anesthetized with a mixture of ketamine/medetomidine (1.5 and 1.0 mg/kg, respectively; i.p.,) and intracardially perfused with ice‐cold PBS 50 mM; pH 7.4). Sagittal separation of the two hemispheres was performed by following the longitudinal fissure. One hemisphere was fixed in 4% PFA (48 h), transferred in sucrose, and frozen. The other one was dissected to collect the hippocampus and cortex, subsequently snap‐frozen and conserved at −80°C.

### 4.9 Immunohistochemistry

Mouse brain slices (3/mouse) were immunolabeled with anti‐GFAP (1:3500; AB5804 Chemicon Int. Inc., USA) for astrocytes and anti‐Iba1 (1:1000; 019‐19,741 Wako) for microglial cells. Slice were at first incubated with H_2_O_2_ (1%) for 10 min followed by 1 h incubation at 4°C with blocking solution (3% NGS + 0.4% Triton X‐100) then overnight with primary antibodies. After incubation with the appropriate biotinylated secondary antibodies (1:200; Vector Laboratories), immunostaining was developed using the avidin‐biotin kit (SP‐2001; Vector Laboratories) and diaminobenzidine (D8001; Sigma‐Aldrich, Italy) as chromogen.

### 4.10 Nissl staining

Twenty‐μm slices were collected with a Leica cryostat on gelatin‐coated slides for a total thickness of: 2.220 μm, from bregma 2.70 to 0.48 (motor cortex); 1960 μm, from bregma −2.56 to −4.52 (whole cortex, striatum, and hippocampus), selecting one slice over every other 5. After 2‐day drying and a 1‐min wash in H_2_O, slices were dehydrated with EtOH (70%, 95%, 100%, 5‐min each), until xylene (5‐min). Slices were processed backward until cresyl violet solution (3‐min), followed by H_2_O + 70% EtOH washes and 3‐min in EtOH/acetic acid. Following 100% EtOH and xylene (5‐min/each), the slices were covered with a glass microcover (Prestige) using DPX mountant.

### 4.11 Image analysis

Quantitative analyses were done by an operator blind to genotype and normalizing on the quantified area. Area of selection for image analysis was the whole hippocampus and/or the whole cortex of a single hemisphere. We analyzed 3, 20‐μm‐thick slices/mouse. Brain images were acquired using the Olympus Virtual Stage microscope. The brain area of interest was selected in bright field, and the immunoreactivity for each specific marker was quantified by applying dedicated homemade macros through Fiji software (Schindelin et al., 2012).

### 4.12 Bulk RNA‐sequencing and bioinformatic analysis

RNA was extracted using TRIzol® (Invitrogen) following the manufacturer's instructions. Nanodrop One C (ThermoFisher) was used for RNA quantification and quality checking. Libraries were prepared using the CORALL Total RNA‐Seq Library Prep Kit (Lexogen), and rRNA was depleted for rRNA ì using RiboCop V1.3 (Lexogen). The quality of libraries was analyzed using 4200 Tape Station with a “DNA High sensitivity” assay (Agilent) and quantified using High Sensitivity dsDNA assay with a Qubit device (Life Technologies). Sequencing was performed using Illumina NextSeq 500, and FastQ files were generated via Illumina bcl2fastq2, version 2.17.1.14 (http://support.illumina.com/downloads/bcl‐2fastq‐conversion‐software‐v217.html), starting from raw sequencing reads produced by Illumina NextSeq sequencer. The raw data obtained from the RNAseq analysis are deposited in the Gene Expression Omnibus repository [accession number GSE270589]. Raw FASTQ files were processed by Unique Molecular Identifiers (UMI) extraction, trimming, alignment, and quality control steps. As CORALL libraries contain N12 UMI at the start of Read 1, in the first step UMI were removed through UMI tools software. Then, adapter sequences, poly(A) sequences at the 3′ end of Read 1 and poly(T) sequences the 5′ end of Read 2, were trimmed through cutadapt software. After UMI extraction and trimming, trimmed reads were aligned through STAR using Gencode Release M29 (GRCm38) as a reference mouse genome. Gene and transcript abundance were computed using FeatureCounts software, with the “stranded forward” option. Differential expression analysis was performed using R package DESeq2 considering different brain regions (i.e., hippocampus and cortex) as covariates in the statistical model (Love et al., 2014). Genes were considered differentially expressed and retained for further analysis with |log_2_(condition sample/control sample)| ≥ 1 and a FDR ≤ 0.1. The R software was used to generate heatmaps (heatmap.2 function from the R ggplots package), PCA plot (prcomp function from the R ggplots package), and Volcano plots. Moreover, a GSEA for the conditions considered was performed by exploiting the clusterProfiler R package (Wu et al., 2021). The MetaboAnalyst 6.0 webtool was used to cross‐reference and perform combined enrichment analysis for metabolomic and transcriptomic analysis (Pang et al., 2024).

### 4.13 Metabolomic

A targeted quantitative approach using combined direct flow injection and liquid chromatography (LC) tandem mass spectrometry (MS/MS) (AbsoluteIDQ 180 kit; Biocrates, Innsbruck, Austria) was employed for the metabolomics analysis of plasma saples. The assay quantifies 186 metabolites from six analyte groups: acylcarnitines, amino acids, biogenic amines, hexoses (sum of hexoses), glycerophospholipids, and sphingomyelins. The method combines derivatization and extraction of analytes with selective mass spectrometric detection using multiple reaction monitoring (MRM) pairs as previously described (Caiola et al., 2016). Multivariate analysis and hierarchical clustering heatmaps were performed using MetaboAnalyst tool v 6.0 (https://www.metaboanalyst.ca/home.xhtml). Wilcoxon–Mann–Whitney test was used to assess statistically significant differences between groups and Spearman correlation to test the correlation between brain parameters and plasma metabolites (GraphPad Prism 10.1.1).

## AUTHOR CONTRIBUTIONS

PL, LA, ES, GC, SL executed in vivo experiments. LD, LA executed histological analysis, image acquisition and signal quantification, and statistical analyses. LB provided all metabolomics data and analyses, RP supervised metabolomics studies. SG, OP executed transcriptomic experiments; LM, SC provided all data on transcriptomics analyses; CC supervised transcriptomics studies. EM executed MRI experiments, and LD provided MRI volumetric data and statistical analyses. CB, GF designed the study. CB coordinated the study, wrote the manuscript, and prepared the figures. LB, LM, SC contributed to write the paper. GF, CC, and AG revised the manuscript.

## CONFLICT OF INTEREST STATEMENT

We have no conflicts of interest to declare.

## Supporting information


Figure S1.

Table S1.

Table S2.

Table S3.

Table S4.

Table S5.

Table S6.

Table S7.

Table S8.

Table S9.

## Data Availability

The data supporting the findings of this study are available upon reasonable request to the corresponding author.
