# Supplementary figures and images for: Transcriptomic and metabolomic changes might predict frailty in SAMP8 mice
Source: Aging Cell. 2024 Jul 3;23(10):e14263. doi: 10.1111/acel.14263 (PMC11464142; doi:10.1111/acel.14263)

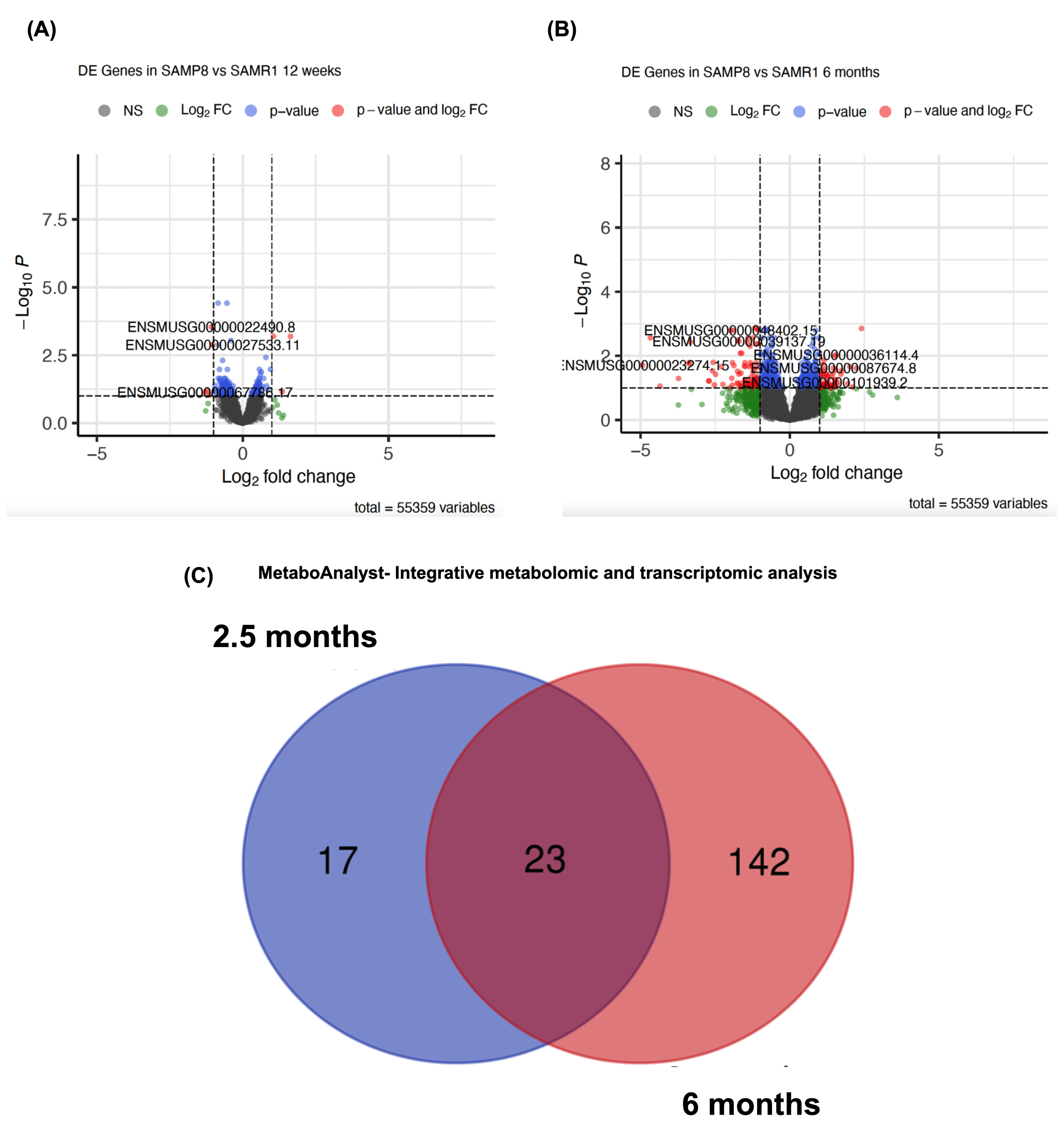

Supplement: Supplementary file 1 — Figure S1. [file ACEL-23-e14263-s002.tiff]
