# Supplementary material for: Transcriptomic and metabolomic changes might predict frailty in SAMP8 mice
Source: Aging Cell. 2024 Jul 3;23(10):e14263. doi: 10.1111/acel.14263 (PMC11464142; doi:10.1111/acel.14263)
Supplement: Supplementary file 9 — Table S8. [file ACEL-23-e14263-s004.docx]

| **Correlation pair SAMR1-SAMP8** | | **r** |
| --- | --- | --- |
| **Cognitive indicators** | **Metabolites** | **2.5M** |
| NORT | Histamine | 0.90 |
|  | PC ae C32:2 | 0.89 |
|  | PC ae C36:4 | 0.81 |
|  | SM (OH) C14:1 | 0.80 |
|  | SM C18:0 | 0.91 |
|  | PC aa C34:2 | 0.89 |
|  | PC ae C34:3 | 0.83 |

**Supplementary Table 8 A. Significant (p<0.05, r>0.8) correlation pair between plasma metabolites and dementia-related cognitive indicators**

| **Correlation pair SAMR1-SAMP8** | | **r** |
| --- | --- | --- |
| **Neuropathological indicators** | **Metabolites** | **2.5M** |
| GD | C14:1 | -0.93 |
|  | C16:1 | -0.86 |
|  | C18:1 | -0.81 |
|  | PC aa C32:0 | -0.86 |
|  | PC aa C34:4 | -0.91 |
|  | PC aa C32:1 | -0.86 |
| STRIATUM | Spermidine | 0.83 |
|  |  |  |
|  |  | **6M** |
|  | Leucine | 0.80 |
| NISSL CTX TOT | C18:1 | 0.96 |
| NISSL STRIATO | Ac-Orn | -0.81 |
|  | Alanine | -0.81 |
|  | Methionine | -0.81 |
|  | C14:1 | 0.96 |
|  | C16:1 | 0.97 |
|  | C18:1 | 0.81 |
|  | C18:2 | 0.92 |
|  | PC ae C34:3 | 0.83 |
|  | PC ae C34:2 | 0.81 |
| GFAP HP | C3-DC (C4-OH) | 0.85 |
|  |  |  |
|  |  | **9M** |
| IBA HP | PC aa C34:1 | 0.81 |
|  | PC aa C34:3 | 0.83 |
|  | PC aa C38:6 | 0.81 |
| IBA CTX | C4 | 0.85 |
|  | lysoPC a C16:1 | 0.92 |
|  | lysoPC a C18:1 | 0.92 |
|  | lysoPC a C18:2 | 0.82 |
|  | lysoPC a C20:3 | 0.83 |
|  | PC aa C32:1 | 0.85 |
|  | SM (OH) C14:1 | 0.83 |
|  | SM (OH) C16:1 | 0.97 |
|  | SM C16:1 | 0.81 |
|  | C16:1 | 0.88 |
| GFAP HP | C18:1 | 0.81 |
|  | PC ae C44:5 | 0.83 |
|  | C16:1 | 0.90 |
|  |  |  |

**Supplementary Table 8 B. Significant (p<0.05, r>0.8) correlation pair between plasma metabolites and dementia-related neuropathological indicators**

**Supplementary Table 8C. Significant (p<0.05, r>0.8) correlation pair between plasma metabolites and dementia-related brain structural indicators**

| **Correlation pair SAMR1-SAMP8** | | **r** |
| --- | --- | --- |
| **Brain structural indicators** | **Metabolites** | **9M** |
| Whole Brain | t4-OH-Pro | -0.87 |
|  | C3-DC (C4-OH) | -0.85 |
|  | lysoPC a C20:3 | 0.81 |
|  | PC ae C38:0 | 0.91 |
|  | PC ae C42:3 | 0.93 |
|  | SM (OH) C14:1 | 0.84 |
|  | PC aa C32:3 | 0.83 |
|  | SM C18:0 | 0.95 |
| Cerebral Cortex | t4-OH-Pro | -0.85 |
|  | C3-DC (C4-OH) | -0.83 |
|  | lysoPC a C20:3 | 0.86 |
|  | PC ae C38:0 | 0.89 |
|  | PC ae C42:3 | 0.89 |
|  | SM (OH) C14:1 | 0.86 |
|  | SM C18:0 | 0.91 |
| Hippocampus | lysoPC a C16:1 | 0.87 |
|  | lysoPC a C20:3 | 0.93 |
|  | PC ae C42:3 | 0.83 |
